# Towards a positive postnatal experience in Sub-Saharan African countries: the receipt of adequate services during the immediate postpartum period: a multilevel analysis

**DOI:** 10.3389/fpubh.2023.1272888

**Published:** 2023-12-13

**Authors:** Aklilu Habte, Aiggan Tamene, Legesse Tesfaye

**Affiliations:** School of Public Health College of Medicine and Health Sciences, Wachemo University, Hosanna, Ethiopia

**Keywords:** postnatal care, adequate postnatal care, multilevel analysis, determinants, Sub-Saharan Africa

## Abstract

**Background:**

Existing studies in the Sub-Saharan African (SSA) region have focused mainly on the frequency of postnatal visits, with little emphasis on the adequacy of care provided during visits. Hence, this study aimed to investigate the magnitude of receiving an adequate number of World Health Organization-recommended contents of care during the immediate postpartum visit, and its predictors in SSA countries.

**Methods:**

The appended women file of the most recent (2016–2021) standardized Demographic and Health Survey report of eighteen Sub-Saharan African countries with a weighted sample of 56,673 women was used for the study. The influence of each predictor on the uptake of adequate postnatal care has been examined using multilevel mixed-effects logistic regression. Significant predictors were reported using the adjusted odds ratio (aOR) with their respective 95% confidence intervals (95% CI).

**Results:**

The pooled prevalence of adequate postnatal care service uptake was found to be 42.94% (95% CI: 34.14, 49.13). Living in the southern sub-region (aOR = 3.08 95% CI: 2.50, 3.80), institutional delivery (aOR = 3.15; 95% CI: 2.90, 3.43), early initiation of ANC (aOR = 1.74; 95% CI: 1.45, 2.09), quality of antenatal care (aOR = 1.59; 95% CI: 1.42, 1.78), Caesarean delivery (aOR = 1.59; 95% CI: 1.42, 1.78), autonomy in decision-making (aOR = 1.30; 95% CI: 1.11, 1.39), high acceptance toward wife beating attitude (aOR = 0.83; 95% CI: 0.73, 0.94), and reading newspapers (aOR = 1.37; 95% CI: 1.21, 1.56) were identified as predictors of receiving adequate postnatal services during the immediate postpartum period.

**Conclusion:**

The findings revealed low coverage of adequate postnatal care service uptake in the region. The Federal Ministry of Health and healthcare managers in each country should coordinate their efforts to develop interventions that promote women’s empowerment to enhance their autonomy in decision-making and to reduce attitudes towards wife beating. Healthcare providers ought to strive to provide skilled delivery services and early initiation of antenatal care.

## Introduction

The days and weeks following childbirth, known as the postnatal period, are essential in the lives of both mothers and newborns (1). This is a critical time for women and newborns. Major physical, social, and emotional changes occurred during this period; nonetheless, it is typically the most neglected period on the maternal and childcare continuum (2). The majority of maternal and neonatal deaths occur within the first 42 days, with over half of postpartum maternal deaths occurring within the first 24 h (3) and 66% occurring within the first week (4). In 2013, 2.8 million newborns died in their first month of life, with one million dying on the first day (5).

Despite an increase in the number of maternal and child health programs and global maternal health efforts, the risk of both maternal and neonatal death after childbirth remains high in developing countries (6). Almost all (99%) maternal and newborn mortality occur in developing countries, with South Asia and Sub-Saharan Africa (SSA) exhibiting the highest rates. Tragically, it is projected that 390 women will die in childbirth for every 100,000 live births in Sub-Saharan Africa by 2030 (7). This is more than five times the 2030 SDG target of fewer than 70 maternal deaths per 100,000 live births, and significantly higher than the European average of thirteen deaths per 100,000 live births in 2017 (7, 8). The increment in morbidity and death is typically the result of a lack of appropriate, suitable, or timely care during that period (9).

Preterm birth, birth asphyxia, and sepsis account for more than 75% of all neonatal deaths, while postpartum haemorrhage and infections account for 80% of maternal deaths (10). All of these are avoidable and manageable causes if high-quality care is provided during the early and early postpartum period (10, 11). Postnatal Care (PNC) is a constellation of services provided to the mother and newborn throughout the first 42 days of life, starting as soon as the expulsion of the placenta (11). It is crucial for the woman and her newborn to recognize and treat health problems during the postpartum period, as well as to provide information to the mother about her and her newborn’s health (3). In addition, the services provided during this period have significance in preventing physical and mental deterioration in post-natal mothers. An array of services provided during this pivotal period are promoting healthy behaviours such as breastfeeding, assessment and treatment of maternal and neonatal danger signs via proper cord examination and cleaning, measurement of newborn body temperature, and referral service to advanced care (11–13).

Integration of PNC with the existing health system is a practicable and viable solution for reducing the high burden of preventable maternal and neonatal morbidity and achieving maternal health-related Sustainable Development Goal (SDG) targets (14). Despite this only 48% of women in SSA had skilled delivery services, less than half received PNC contact within 2 days of childbirth, and only 13% of women who gave birth at home received postpartum visits within 2 days of delivery (4, 12). This means that PNC is one of the most neglected services among all reproductive and child health interventions.

Through a technical consultation process, the World Health Organization (WHO) recently updated global recommendations on the timing and content of PNC for women and newborns with a particular emphasis on resource-constrained settings in low-and middle-income countries (LMIC) (11). Regardless of where childbirth takes place, the WHO recommends that all mothers and newborns receive four postpartum visits: the first within the first 24 h, followed by the second, third, and fourth visits within 3–4, 7–14, and 42 days following child birth, respectively (11). Receiving adequate postnatal care, which includes clinical and non-clinical maternal and newborn care, health promotion, and health systems interventions, is one of the quality indicators in maternal health service provision and it plays a significant role in lowering preventable deaths in the postpartum period (15). However, existing studies in the SSA region (16–18) have focused mainly on the frequency of PNC visits, with little emphasis on the contents of care provided during visits.

Hence, this study aimed at investigating the magnitude of receiving an adequate care during PNC visits, and its predictors at the individual and community levels. The findings of this study will help policymakers, programme managers, and service providers in devising intervention plans by acting on individual and community-level variables that hinder the uptake of basic PNC packages. This, in turn, will reduce maternal and newborn deaths, enhance health outcomes, and promote community-based health systems and women-centered maternity care in the region.

## Methods

### Data source, population, and study period

The most recent Demographic and Health Surveys (2016–2021) data from eighteen Sub-Saharan African countries were used for this study. The DHS is a nationwide representative survey that collects data relating to basic maternal and child health indicators. The current dataset has been created by appending women files (IR) together, which is proper for most woman-level analyses based on fertility, contraception, and prenatal, intrapartum, and postpartum service uptake. The current study covered countries having recent standardized DHS reports from 2016 to 2021 containing complete information on the relevant variables. Only women of reproductive age who had at least one PNC visit were included. A total of a weighted sample of 56,673 women of reproductive age who gave birth in the last 2 years preceding the survey were included in the analysis (Table 1).

**Table 1:** List of the SSA countries included in the analysis with their respective weighted sample size, 2016–2021.

Regions and countries	DHS Year	Weighted sample size [*n*()]
Central Region	2016–2018	5,718 (10.1)
Angola	2016	2,005 (3.5)
Cameroon	2018	3,713 (6.6)
Eastern Region	2016–2021	21,225 (37.4)
Burundi	2017	761 (1.3)
Ethiopia	2016	632 (1.1)
Madagascar	2021	2,989 (5.3)
Malawi	2017	5,938 (10.5)
Rwanda	2020	1,138 (2.1)
Uganda	2016	2,280 (4.0)
Zambia	2018	4,633 (8.2)
Mauritania	2020	2,854 (5.0)
Western Region	2018–2020	27,250 (48.1)
Benin	2018	1,728 (3.0)
Gambia	2020	5,827 (10.3)
Guinea	2018	1,923 (3.4)
Liberia	2021	1,983 (3.6)
Mali	2018	3,359 (5.9)
Nigeria	2018	9,100 (16.0)
Sierra Leone	2020	3,329 (5.9)
Southern region	2016	2,478 (4.4)
South Africa	2016	2,478 (4.3)
Total	2016–2021	56,673 (100.0)

### Data collection tool and procedures

Using standardized questionnaires developed in each country’s official language, the data were collected through face-to-face interviews with trained data collectors. To select study participants, the DHS employs a two-stage stratified sampling technique. The first stage entailed selecting enumeration areas (EAs) and listing households within those EAs using the most recent population and housing census data from each country as a sampling frame. In the second stage, households were selected using an equal probability sampling technique. The Demographic and Health Survey Sampling and Household Listing Manual developed by ICF International goes over the thorough sampling technique implemented during DHS (19).

### Measurement of variables of the study

The outcome variable of the current study was the receipt of adequate PNC services during the immediate postpartum period. Per WHO recommendation, the following five components were used to assess the receipt of adequate PNC service: the newborn’s cord was inspected, the newborn’s temperature was measured, the mother was counseled about newborn danger signs, breastfeeding counseling, and an observation of a breastfeeding session (11). The services obtained during the immediate PPP were assessed using the following questions: “During the first 2 days, did the health provider examine the newborn’s cord?,” “During the first 2 days, did the health provider measure the newborn’s temperature?…” If a mother said “Yes,” the response was labeled as 1, otherwise as 0. During the same childbirth, a single mother can respond that cord care was given to her newborn or that her newborn’s temperature was taken numerous times, but all response was recorded as a single service. A composite index of PNC service items was generated based on the responses, which is a tally of the number of key elements of PNC received. The variable had a minimum value of zero, indicating that none of the above-mentioned services were received, and a maximum value of five, indicating that the woman received all services during PPP. When a woman got all five service items, she was deemed to have received adequate services during PPP (15).

### Explanatory variables

Following a review of relevant studies, individual and community-level factors that are supposed to influence the receipt of adequate postnatal care service were extracted from the data (Table 2).

**Table 2:** List of factors that affect the uptake of adequate PNC services during the immediate postpartum period in SSA countries, 2016–2021.

Individual-level factors		
Variables	Description	Category
Maternal age	The respondent’s age, expressed in years, at the time of the survey	15–24^*^ 25–34, 35 and above
Women educational attainment	Percent distribution of women ages 15–49 by the highest level of schooling attended or completed	No education^*^, Primary Secondary Higher
Occupational status	Percent distribution of women and men employed in the 12 months preceding the survey by occupation	Unemployed^*^ Employed
Family size	Number of household members at the time of data collection	≤5 >5^*^
Sex of head of Household	Percent distribution of households by sex of head of household	Female Male^*^
Wealth index	Calculated using easy-to-collect data on a household’s ownership of selected assets, such as televisions and bicycles; materials used for housing construction; and types of water access and sanitation facilities	Richest Richer Middle Poorer Poorest^*^
Health insurance	Percentage of women aged 15–49 with any type of health insurance scheme	Yes, No^*^
Having a mobile phone	Percentage of women age 15–49 who own a mobile phone	Yes, No^*^
Had bank account	Percentage of women aged 15–49 who use an account in a bank or other financial institution	Yes No^*^
Access to Internet	Percentage of women aged 15–49 who have ever used the internet	1. Yes 2. No^*^
Media exposure	Number of women age 15–49 who are exposed to specific media with various frequencies: read a newspaper, watch television, and listen to radio	Not at all^*^ Less than once a week At least once a week
Problems in accessing healthcare	A number of women aged 15–49 reported that they have serious problems in accessing health care for themselves when they are sick: getting permission to go for treatment, getting money for treatment, and distance to the health facility	Not a big problem A big problem^*^
Total children ever born	Percentage of women with a specified number of children ever born at the time of the survey	No^*^ One Two to four Five and more
Pregnancy status during last childbirth	Percent distribution of births to women aged 15–49 in the 5 years preceding the survey, including current pregnancies, by planning status of the birth – (i) wanted then, (ii) wanted later, or (iii) not wanted at all	Un planned (ii & iii) Planned(i)^*^
Visit a health facility	Number of women aged 15–49 who visited a health facility in the preceding 12 months and who discussed family planning	Yes No
Place of delivery	Percent distribution of live births in the past 5 years by place of delivery	Health facility Home^*^
Timing of ANC	Percentage of women with a birth in the last 5 years, distributed by the number of months pregnant at the time of first antenatal care visit for the most recent birth	1st trimester 2nd trimester 3rd trimester
Frequency of antenatal care	Percentage of women with a birth in the last 5 years, distributed by number of antenatal care visits for most recent birth	No visit One visit 2–3 visits 4 and more visits
Quality of ANC	Percentage of women with a birth in the past 5 years who took all of the following five items of care: iron tablets or syrup, intestinal parasite drugs, their blood pressure measured, a urine sample taken, and, a blood sample taken	Yes No^*^
Delivery by CS	Percentage of live births in the 5 years preceding the survey delivered by cesarean section	Yes, No^*^
Provider of PNC service	All categories of health providers are considered, including community health workers and traditional birth attendants: By trained health care providers(medical personnel, doctor, nurse, midwives, Auxiliary midwives or community nurse attendants, others), by traditional birth attendants, or by community health workers (CHWs)	Trained HCPs CHWs TBAs^*^
The overall attitude towards wife beating^a^	A percentage of women aged 15–49 agreed or disagreed that a husband is justified in hitting or beating his wife for five specific reasons: burning food, arguing with him, going out without telling him, neglecting the children, refusing to have sexual intercourse with him, at least one reason	Low Moderate High^*^
Autonomy in decision-making^b^	The aggregate distribution of currently married women aged 15–49 who usually makes decisions about their own health care, large household purchases, and visits to family or relatives	Low^*^ Medium high
Community-level factors		
Residence	The area where respondents lived when the survey was conducted	Urban rural^*^
Region	Central Region-Angola, Cameroon, Eastern Region-Burundi, Ethiopia, Madagascar, Malawi, Rwanda, Uganda, Zambia, and Mauritania Western Region-Benin, Gambia, Guinea, Liberia, Mali, Nigeria, and Sierra Leone Southern-South Africa	Central Region Eastern Region Western Region Southern^*^

^*^Reference category.

a Attitude toward wife beating: acceptance of wife beating was measured by using five items: (i) beating justified if she neglects children, (ii) beating justified if she argues with her husband, (iii) beating justified if she refuses to have sex, (iv) beating justified if she goes out without the permission of her husband, and (v) beating justified if she burns foods. The response categories for each item were (i) no, (ii) yes, and (iii) do not know. Response (i) was given a value of 0, indicating that it was not accepted, and the other responses were given values of 1, indicating that they agreed on wife beating. Responses to those five items were combined to produce a composite score, which ranges from 0 to 5. A lower score on this indicator is seen as implying a better sense of entitlement and self-esteem, as well as a higher level of women’s status (20). Lastly, a composite index was divided into three categories: low, middle, and high for scores at “0 to 2,” “3 to 4,” and “5,” respectively (21, 22).

b Overall decision-making power: it was assessed based on responses to questions on who makes ultimate decisions for the family when it comes to big purchases for homes, visits to family or relatives, and health care. The response categories were (i) respondent alone, (ii) respondent and husband/partner, (iii) husband/partner alone, (iv) someone else, and (v) others. For each question, (i) or (ii) responses were assigned a value of one, indicating high decision-making power, while other responses were assigned a value of zero, indicating low power. The responses to the three aspects of decision-making power were summed to produce a composite score ranging from 0 to 3. This index is linked to women’s empowerment and indicates their level of decision-making capability in areas that affect their own lives. Finally, a composite score was split into three categories: low, middle, and high for “0 to 1,” “2,” and “3” scores, respectively (22, 23).

### Data management and statistical analyses

The most recent DHS reports from 18 SSA countries were appended, recoded, cleaned, and analyzed using STATA version 16.0. Weighting was done to ensure the statistical representativeness of the survey and to come up with robust statistical estimates. Descriptive statistics such as frequencies, percentages, and the mean of the covariates across the outcome variables were computed to figure out the characteristics of the respondents. Using random meta-analysis, the pooled prevalence of adequate PNC service usage was estimated.

### Multilevel analyses

Because the DHS data is hierarchical, it defied assumptions of ordinary regression such as independence of observations and equal variance. This implies that the between-cluster variability needs to be considered, and so advanced models like multilevel mixed-effect regression analyses were employed to handle those issues. Running a multilevel analysis upon such hierarchical data allows for minimal biased parameter estimates.

### Statistical model building and selection

#### Fixed effects (measures of association)

Since a receipt of adequate PNC is an outcome variable that is categorized as Yes = 1 or No = 0, a multilevel mixed-effect logistic regression model was fitted. First, a multilevel bivariable logistic regression analysis was carried out to examine the relationship between each predictor and receipt of adequate PNC, and variables with value of *p* < 0.25 were entered into a multilevel multivariable mixed-effect logistic regression. In the multilevel multivariable logistic regression model, adjusted odds ratios (aOR) with a 95% CI were used to identify significant predictors of adequate PNC uptake. The Variance Inflation Factor (VIF) was estimated to check for the presence of multicollinearity between the variables, and it found that there was no significant multicollinearity (the VIF ranged from 1.02 to 6.40, with a mean of 1.62).

#### Random effects (measures of variation)

Four distinct models were fitted to identify significant predictors. Model-I is a null model (with no covariates), Model II (contains only individual-level factors), Model-III (contains only community-level factors), and Model-IV (a full model comprising both individual-and community-level factors).

Intra-class correlation coefficient (ICC), median odds ratio (MOR), and proportional change in variance (PCV) metrics were computed for measures of variation.

ICC is a measure of the degree of heterogeneity of not having full PCV between clusters, and it was determined using:


$$ICC=\frac{\mathrm{var}\left(b\right)}{Var\left(b\right)+Var\left(w\right)}=\frac{\mathrm{var}\left(b\right)}{Var\left(b\right)+3.29}$$

where Var(*b*) is the estimated variance each model and Var(*w*) is a predicted individual variance component, which is *π*2/3 ≈ 3.29.

The PCV was used to evaluate the contribution of individual-and/or community-level factors to the overall variation in the null model, and it was determined by:


$$PCV=\frac{\left(Va-Vb\right)}{Va}*100$$

where, *Va* is the variance of the initial model (null model) and *Vb* = variance of the subsequent models (models 2, 3, and 4).

### Model fitness

Model comparability and fitness were based on Deviance = −2 * Log Likelihood (LL), Akaike’s information criteria (AIC), and Schwarz’s Bayesian Information Criterion (BIC). The model with the lowest AIC and deviance was selected for the final interpretation of the findings.

## Results

### Socio-demographic and obstetric characteristics of respondents

This study was based on a weighted sample of 56,673 women of reproductive age in SSA countries who gave birth within 2 years preceding the survey. Nearly half (48.1%) and more than one-third (37.4%) of respondents were from western and eastern sub-regions, respectively. Nigeria, Malawi, and Gambia all contributed considerably to the sample size, with 16.0, 10.5, and 10.3%, respectively. The mean (±SD) age of respondents was 29.17(±7.21) years, with the majority (46.3%) being between the ages of 25 and 34. The majority (58.2%) of respondents were rural residents. There was a statistically significant difference in the receipt of adequate PNC across women’s educational levels, sub-regions, place of residence, sex of head of household, and the total number of children ever born (*p* < 0.001) (Table 3).

**Table 3:** The receipt of adequate services during immediate postpartum period across socio-demographic and obstetric characteristics of women in SSA countries, 2016–2021.

Variable categories	Weighted sample size (*N* = 56,673)	Received adequate PNC service (*N* = 24,430)	
	[*n*()]	Yes [*n* (%)]	COR (95% CI)
Sub-Regions			
Central	5,719 (10.1)	2,331 (9.5)	1
Eastern	21,226 (37.4)	8,651 (35.4)	0.93 (0.81, 1.07)
Western	27,250 (48.1)	11,697 (47.9)	1.03 (0.89, 1.19)
Southern	2,478 (4.4)	1,751 (7.2)	3.70 (3.05, 4.50)^*^
Current Age			
15–24	16,539 (29.2)	7,073 (29.0)	1
25–34	26,261 (46.3)	11,338 (46.4)	1.00 (0.94, 1.07)
35–49	13,874 (24.5)	6,019 (24.6)	1.01 (0.95, 1.08)
Educational status			
No education	16,569 (29.2)	6,265 (25.6)	1
Primary	17,726 (31.3)	7,004 (28.7)	1.07 (0.99, 1.15)
Secondary	18,861 (33.3)	9,251 (37.9)	1.61 (1.49, 1.74)^*^
Higher	454 (6.2)	1,910 (7.8)	1.99 (1.73, 2.29)^*^
Occupational status			
Not working	14,667 (25.9)	7,039 (28.8)	1
Had work	42,006 (74.1)	17,391 (71.2)	0.94 (0.79, 1.09)
Residence			
Rural	32,964 (58.2)	13,012 (53.3)	1
Urban	23,709 (41.8)	11,418 (46.7)	1.49 (1.36, 1.62)^*^
Family size			
>5 member	32,323 (57.0)	13,449 (55.0)	1
≤5 member	24,350 (43.0)	10,981 (45.0)	1.12 (1.06, 1.18)^*^
Head of household			
Male	44,039 (77.7)	18,462 (75.6)	1
Female	12,634 (22.3)	5,968 (24.4)	1.25 (1.18, 1.33)^*^
Community Education			
Low	20,291 (35.8)	8,862 (36.3)	1
Moderate	19,399 (34.2)	8,456 (34.6)	0.93 (0.85, 1.03)
High	16,983 (30.0)	7,112 (29.1)	1.11 (0.95, 1.09)
Wealth index combined			
Poorest	10,849 (19.1)	4,029 (16.5)	1
Poorer	11,165 (19.7)	4,252 (17.4)	1.07 (0.98, 1.17)
Middle	11,418 (20.2)	4,710 (19.3)	1.21 (1.10, 1.33)^*^
Richer	11,530 (20.3)	5,462 (22.4)	1.58 (1.42, 1.76)^*^
Richest	11,711 (20.7)	5,977 (24.5)	1.83 (1.64, 2.04)^*^
Community poverty			
Low	20,210 (35.7)	8,891 (36.9)	1.19 (1.08, 1.33)^*^
Moderate	19,374 (34.2)	8,452 (34.6)	1.11 (1.00, 1.22)
High	17,089 (30.1)	7,087 (29.01)	1
Parity			
Nulliparous	379 (0.7)	152 (0.6)	1
Primiparous	13,767 (24.3)	6,490 (26.6)	1.31 (0.95, 1.81)
Multiparous	29,103 (51.3)	12,763 (52.2)	1.15 (0.83, 1.58)
Grand multiparous	13,424 (23.7)	5,025 (20.6)	0.87 (0.63, 1.21)
Total Children ever born			
1	12,900 (22.7)	6,137 (25.1)	1.51 (1.40, 1.63)^*^
2–4	27,516 (48.5)	12,172 (49.8)	1.32 (1.23, 1.41)^*^
≥5	16,257 (28.7)	6,120 (25.1)	1
Pregnancy status			
Wanted	40,732 (71.9)	17,624 (72.5)	1.05 (0.94, 1.16)
Mistimed	12,388 (21.9)	5,303 (21.7)	1.03 (0.92, 1.15)
Unwanted	3,553 (6.3)	1,503 (6.2)	1
Overall acceptance of wife beating			
Low	43,620 (77.0)	19,221 (78.7)	1.43 (1.28, 1.60)^*^
Medium	8,681 (15.3)	3,663 (15.0)	1.35 (1.20, 1.52)^*^
High	4,371 (7.7)	1,546 (6.3)	1

1, reference category; COR, crude odds ratio; ^*^, significant at value of *p* < 0.25.

### Health service-related characteristics of respondents

**Figure 1:**
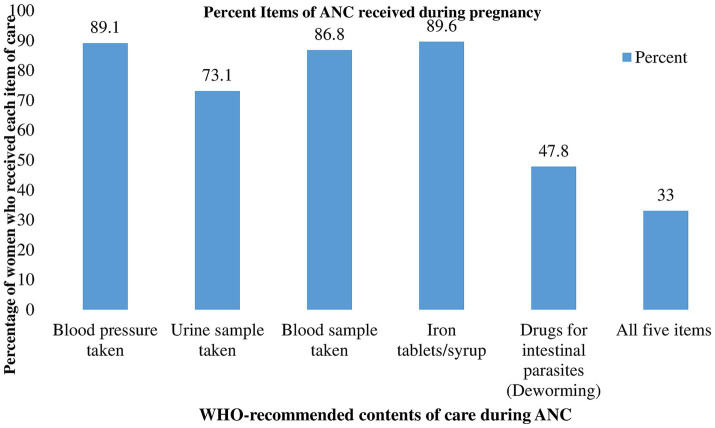
Percentage distribution of the WHO-recommended contents of ANC received during the most recent pregnancy in SSA, 2016–2021.

Almost three-quarters (73.1%) of women had their last childbirth in a health facility. In terms of ANC service utilization, 40.1% of women received their 1st ANC visit during the first trimester, whereas just 6.4% received four or more ANC visits. Only one-third (33.0%) of women received all of the WHO-recommended items of care during their most recent ANC visits (Figure 1). Almost half of all women (47.7%) were non-autonomous in decision-making. Lack of money, distance to health facilities, and inability to get permission have been identified as important barriers to receiving healthcare services by 45.7, 34.4, and 13.6% of women, respectively. Of women, 82.3, 38.2, and 51.2% had never watched television, never read a newspaper, and never listened to the radio, respectively. Only 5.6% of women were enrolled in any type of health insurance scheme (Table 4).

**Table 4:** The receipt of adequate services during immediate postpartum period across health service-related characteristics women in SSA countries, 2016–2021.

Variable categories	Weighted sample size (*N* = 56,673)	Received adequate PNC service (*N* = 24,430)	
	[*n* (%)]	Yes [*n* (%)]	COR (95% CI)
A recent visit to a health facility			
Yes	41,254 (72.8)	18,030 (73.8)	1.11 (1.04, 1.18)^*^
No	15,419 (27.2)	6,400 (26.2)	1
Place of delivery			
Health facility	41,424 (73.1)	21,120 (86.4)	4.03 (3.73, 4.36)^*^
Home	15,249 (26.9)	3,310 (13.6)	1
Timing of ANC			
1st trimester	22,756 (40.1)	11,089 (45.4)	2.85 (2.50, 3.26)^*^
2nd trimester	30,151 (53.2)	12,354 (50.6)	2.05 (1.80, 2.32)^*^
3rd trimester	3,766 (6.7)	987 (4.0)	1
Frequency of ANC			
No visit	3,874 (6.8)	1,358 (5.5)	1
One visit	1,527 (2.7)	482 (2.0)	0.91 (0.74, 1.12)
2–3 visits	13,643 (24.1)	5,074 (20.8)	1.19 (1.02, 1.38)^*^
4 and more visits	37,629 (6.4)	17,516 (71.7)	1.76 (1.52, 2.04)^*^
Quality of ANC			
Yes	18,696 (33.0)	10,582 (43.3)	2.42 (2.26, 2.59)^*^
No	37,977 (67.0)	13,848 (56.7)	1
Delivery by CS			
Yes	3,741 (6.6)	2,421 (9.9)	2.59 (2.33, 2.88)^*^
No	52,932 (93.4)	22,009 (90.1)	*
Autonomy in decision-making			
Low	27,035 (47.7)	11,242 (46.0)	1
Middle	8,037 (14.2)	3,043 (12.5)	0.83 (0.75, 1.19)
High	21,601 (38.1)	10,144 (41.5)	1.18 (1.10, 1.26)^*^
Distance to a health facility			
Big problem	19,506 (34.4)	7,677 (31.4)	1
Not a big problem	37,167 (65.6)	16,753 (68.6)	1.25 (1.18, 1.34)^*^
Money needed for treatment			
Big problem	25,901 (45.7)	10,188 (41,7)	1
Not a big problem	30,772 (54.3)	14,242 (58.3)	1.34 (1.26, 1.43)^*^
Get permission			
Big problem	7,711 (13.6)	3,278 (13.4)	1
Not a big problem	48,962 (86.4)	21,152 (86.6)	1.02 (0.93, 1.12)
Reading newspaper			
Not at all	46,654 (82.3)	18,906 (77.4)	1
Less than once a week	5,938 (10.5)	3,059 (12.5)	1.57 (1.42, 1.72^*^)
At least once a week	4,080 (7.2)	2,465 (10.1)	2.24 (2.02, 2.50)^*^
Listening to a radio			
Not at all	21,664 (38.2)	8,903 (36.4)	1
Less than once a week	13,894 (24.5)	5,727 (23.4)	1.01 (0.93, 1.09)
At least once a week	21,115 (37.3)	9,800 (40.1)	1.23 (1.15, 1.32)^*^
Watching TV			
Not at all	28,974 (51.1)	11,262 (46.1)	1
Less than once a week	9,202 (16.2)	3,940 (16.1)	1.19 (1.10, 1.30)^*^
At least once a week	18,497 (32.7)	9,228 (37.8)	1.60 (1.49, 1.71)^*^
Covered by Health insurance			
Yes	3,151 (5.6)	1,576 (6.5)	1.36 (1.20, 1.54)^*^
No	53,522 (94.4)	22,854 (93.5)	1
Having a mobile phone			
Yes	32,765 (57.8)	15,469 (63.3)	1.52 (1.44, 1.61)^*^
No	23,908 (42.2)	8,961 (36.7)	1
Had bank account			
Yes	8,343 (14.7)	4,499 (18.4)	1.65 (1.51, 1.80)^*^
No	48,330 (85.3)	19,931 (81.6)	1

1, reference category; COR, crude odds ratio; ^*^, significant at value of *p* < 0.25.

### The uptake of adequate PNC in SSA countries

**Figure 2:**
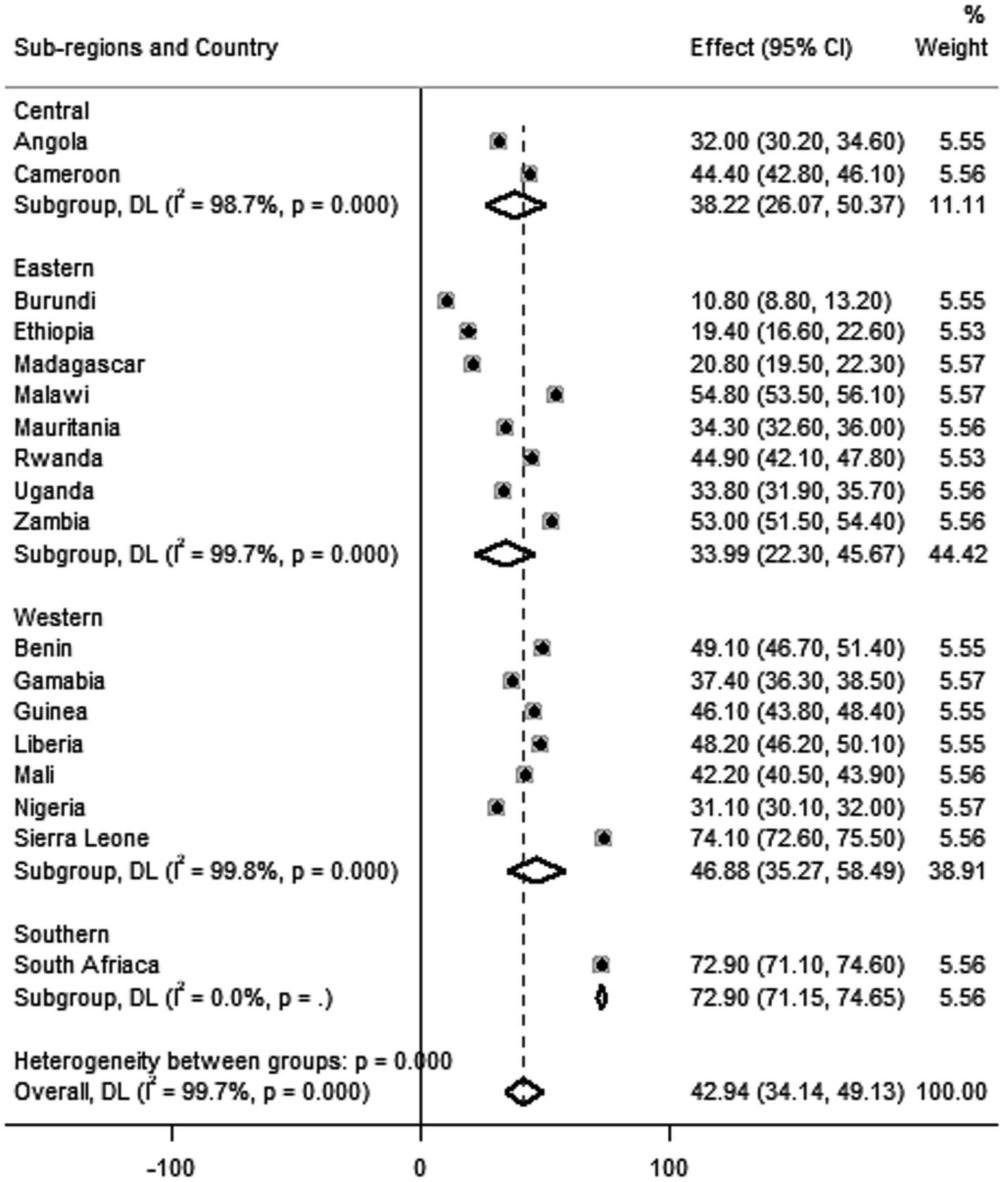
A forest plot depicts the country and regional prevalence of adequate PNC in SSA countries, 2016–2021.

**Figure 3:**
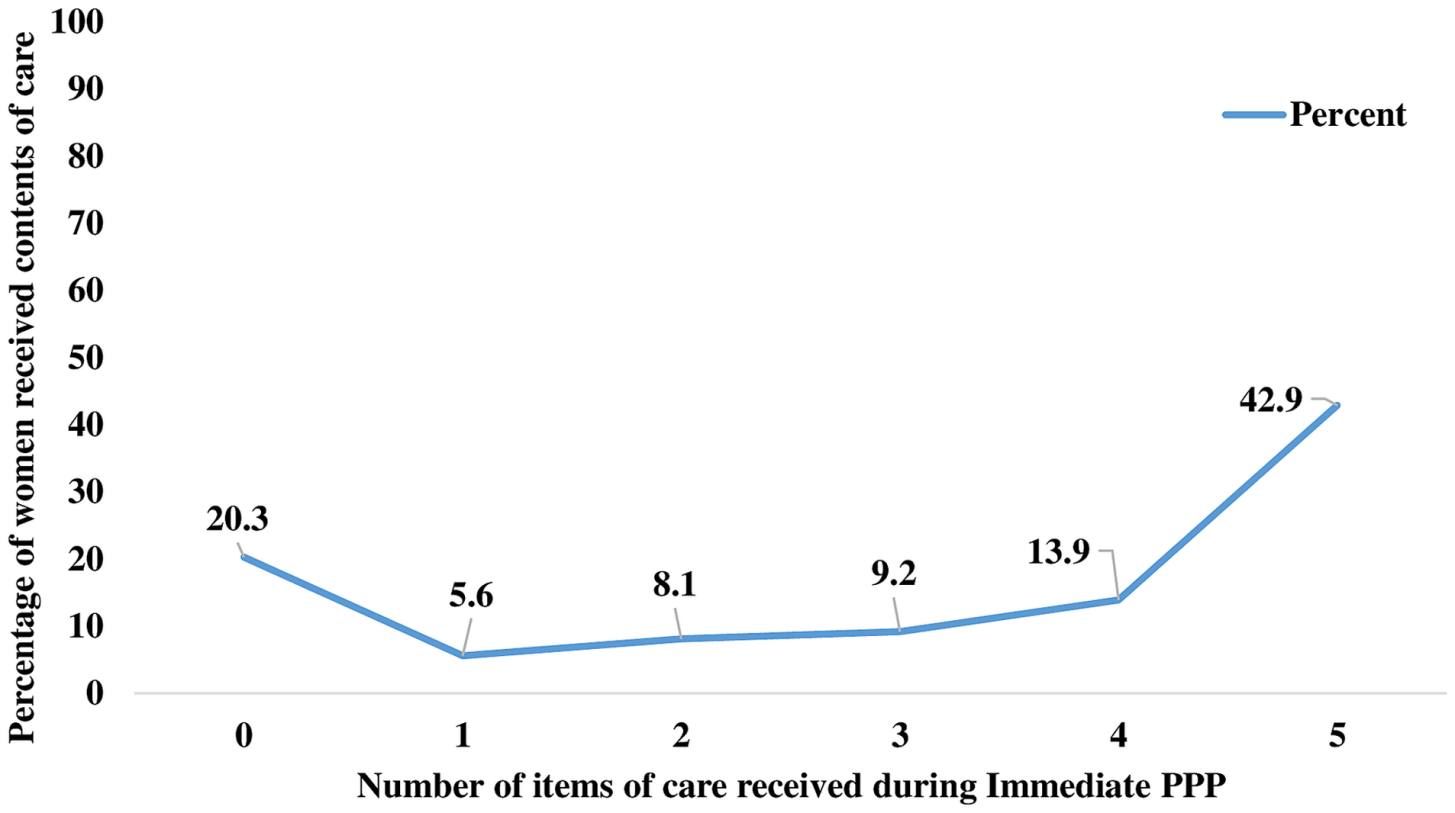
Number of contents of care received by women during the immediate PPP in SSA countries, 2016–2021.

**Figure 4:**
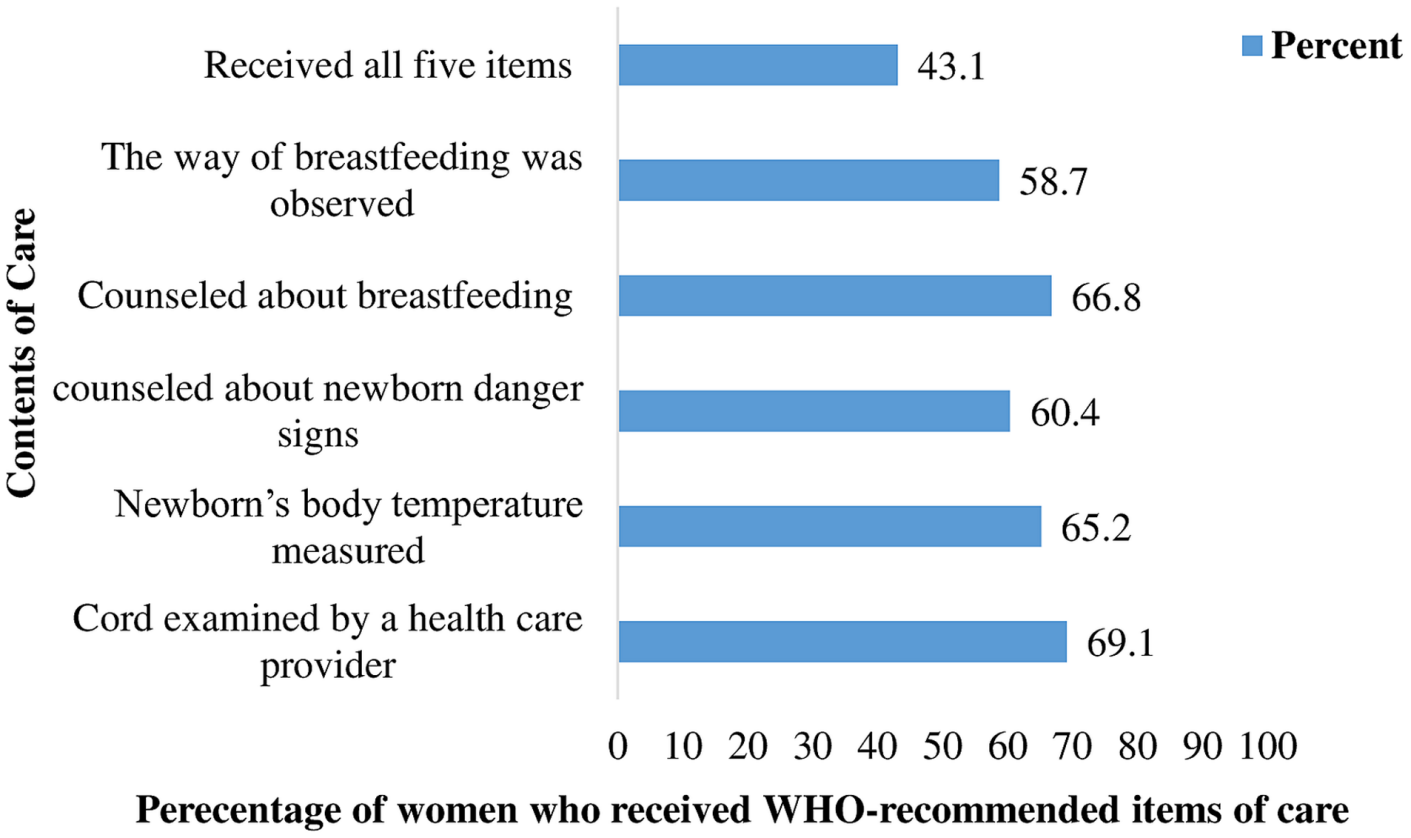
Percentage distribution of each item of care received by women in SSA, 2016–2021.

The pooled prevalence of adequate postnatal care service uptake in SSA countries was 42.94% [95% CI: 34.14, 49.13]. The proportion of women receiving adequate postnatal care services was high in the southern (72.9%) and western (46.88%) sub regions, and lowest in the eastern (33.9%) one. South Africa (72.9%) and Burundi (10.80%) had the highest and lowest proportions of adequate PNC service uptake, respectively (Figure 2). Regarding the individual contents of care, the majority of women (79.9%) received at least one of the five essential contents of care. 9.2 and 13.9% of women received three and four items of care, respectively (Figure 3). The majority of women received cord examination, breastfeeding counseling, and newborn body temperature measurement, with 69.0, 66.8, and 65.2%, respectively, (Figure 4).

### Random effect estimates (measures of variation)

The random effect estimates have been determined by fitting five models (Model I (null model), Model II, Model III, and Model IV). The null model showed significant variability in the likelihood of adequate PNC uptake among SSA countries (*σ*^2^ = 0.49, *p* < 0.001). ICC of 0.129 in the null model, implied that the difference within clusters contributed 12.9% of the total variation in the uptake of adequate PNC services. The proportional change in variance (PCV) in the full model was 23.9%, showing that both individual-and community-level factors explained 40.81% of the observed variation in the null model (Table 5).

**Table 5:** Results of a multivariable mixed-effect multivariable logistic regression to identify the determinants of adequate PNC service uptake in SSA countries, 2016–2021.

Variable categories	Model-I (null model)	Model-II (individual-level)	Model-III (community-level factors)	Model-V (full model)
		aOR (95% CI)	aOR (95% CI)	aOR (95% CI)
Educational status				
Higher		0.88 (0.75, 1.03)		0.87 (0.74, 1.02)
Secondary		0.98 (0.89, 1.08)		0.95 (0.86, 1.04)
Primary		0.87 (0.81, 1.05)		0.94 (0.86, 1.02)
No education		1		1
Family size				
≤5 member		0.98 (0.91, 1.07)		0.97 (0.92, 1.04)
>5 member		1		1
Head of household				
Female		1.18(1.10, 1.26)^*^		0.97 (1.07, 1.22)
Male		1		1
Wealth index combined				
Richest		1.25 (1.11, 1.39)		1.08 (0.94, 1.25)
Richer		1.19 (1.05, 1.36)		1.14 (0.98, 1.28)
Middle		0.97 (0.88, 1.07)		1.02 (0.92, 1.12)
Poorer		1.01 (0.92, 1.10)		1.00 (0.97, 1.03)
Poorest		1		1
Total Children ever born				
≥5		0.80 (0.74, 0.87)		1.02 (0.96, 1.22)
2–4		0.96 (0.89, 1.02)		0.94 (0.87, 1.01)
1		1		1
Place of delivery				
Health facility		3.22 (2.97, 3.50)^*^		3.15(2.90, 3.43)^*^
Home		1		1
Timing of ANC				
1st trimester		1.73(1.45, 2.08)^*^		1.74(1.45, 2.09)^*^
2nd trimester		1.51(1.26, 1.80)^*^		1.53(1.27, 1.83)
3rd trimester		1		1
Frequency of ANC				
4 and more visits		0.98(0.94, 1.03)		1.14(0.97, 1.24)
2–3 visits		0.94(0.89, 1.00)		0.97(0.95, 1.09)
One visit		0.94(0.87, 1.03)		0.93(0.89, 1.01)
No visit		1		1
Quality of ANC				
Yes		2.14(2.00, 2.30)^*^		2.25(2.09, 2.41)^*^
No		1		1
Delivery by CS				
Yes		1.71(1.53, 1.92)^*^		1.59(1.42, 1.78)^*^
No		1		1
Acceptance of wife beating				
High		0.77(0.68, 0.87)^*^		0.83 (0.73,0.94)^*^
Medium		0.82 (0.74, 0.92)^*^		0.86 (0.78, 0.99)
Low		1		1
Autonomy in decision-making				
High		1.16 (1.08, 1.25)^*^		1.30 (1.11, 1.39)^*^
Middle		0.82 (0.75, 1.08)		0.91 (0.83, 0.99)
Low		1		1
Distance to a health facility				
Not a big problem		0.97(0.90, 1.04)		0.97(0.90, 1.05)
Big problem		1		1
Money needed for treatment				
Not a big problem		1.13 (1.05, 1.21)^*^		1.11 (1.04, 1.20)
Big problem		1		1
Reading newspaper				
At least once a week		1.59(1.40, 1.80)^*^		1.37 (1.21, 1.56)^*^
<once a week		1.25 (1.12, 1.38)^*^		1.19 (1.07, 1.33)^*^
Not at all		1		1
Listening to a radio				
At least once a week		1.09 (0.99, 1.19)		0.94(0.87, 1.02)
<once a week		1.02 (0.93, 1.12)		0.87 (0.80, 0.95)
Not at all		1		1
Watching TV				
At least once a week		1.14(0.91, 1.24)		0.98(0.88, 1.08)
<once a week		1.02 (0.96, 1.15)		1.00 (0.98, 1.03)
Not at all		1		1
Covered by Health insurance				
Yes		1.49 (1.28, 1.72)*		1.27 (1.13, 1.53)*
No		1		1
Having a phone				
Yes		1.22 (1.15, 1.30)^*^		1.06 (0.99, 1.13)
No		1		1
Sub-Regions				
Southern			3.69 (3.04, 4.49)^*^	3.06 (2.49, 3.76)^*^
Western			1.04 (0.95, 1.27)	1.16 (1.00, 1.35)
Eastern			1.06 (0.91, 1.22)	1.02 (0.87, 1.18)
Central			1	1
Residence				
Urban			1.24 (1.13, 1.36)^*^	0.93(0.84, 1.04)
Rural			1	1
Random effects				
Variance	0.49	0.39	0.36	0.29
ICC	0.129	0.106	0.098	0.081
AIC	75413.2	68856.4	74320.04	68434.5
BIC	75431.2	69161.4	74373.8	68775.3
PCV	Reference	20.40%	26.53%	40.81%
Model fitness				
Log-likelihood	−37704.6	−34394.2	−37154.0	−34079.2
Deviance (−2LL)	75409.2	68788.4	74310.0	68158.4

1, reference category; aOR, adjusted odds ratio; ^*^, statistically significant at value of *p* < 0.05.

### Fixed effect analysis

#### Determinants of adequate PNC visits: a multilevel-multivariable logistic regression

In a multilevel multivariable logistic regression analysis, variables namely, living in a southern sub-region, low acceptance of wife beating, reading newspapers, place of delivery, the timing of ANC, autonomy in decision-making, quality of ANC, and delivery by CS were identified as significant predictors of receiving adequate PNC services (see Figure 4).

Women residing in the southern region had 3.08 times higher odds of receiving adequate PNC during early PPP than women living in the central region (aOR = 3.08 95% CI: 2.50, 3.80). Timing and quality of ANC were also identified as significant predictors of adequate PNC service uptake. As compared to women who started their first prenatal visit in the third trimester, the odds of receiving adequate PNC were 1.74 (aOR = 1.74; 95% CI: 1.45, 2.09) and 1.53 (aOR = 1.53; 95% CI: 1.27, 1.83) times higher for those who commenced in the first and second trimesters, respectively. Similarly, women who gave birth in health facilities had a 3.15 (aOR = 3.15; 95% CI: 2.90, 3.43) times higher likelihood of receiving adequate PNC than those who gave birth at home. The odds of receiving adequate PNC services increased by 59% (aOR = 1.59; 95% CI: 1.42, 1.78) for women who delivered via CS versus spontaneous vaginal delivery (SVD). Women with high acceptance of wife-beating were 17% less likely to receive adequate PNC service than women with low acceptance (aOR = 0.83; 95% CI: 0.73, 0.94). Women who read newspapers at least once a week had 1.37 times a chance of receiving adequate PNC than women who never read (aOR = 1.37; 95% CI: 1.21, 1.56). Having the highest decision-making autonomy increased the likelihood of receiving adequate PNC service by 30% (aOR = 1.30; 95% CI: 1.11, 1.39) (Table 5).

## Discussion

According to the World Health Organization’s vision for quality of care, countries should shift their focus from focusing solely on coverage of maternal health services to offering essential elements of care during each service delivery (24). The current study used the most recent demographic health surveys to analyze the level of adequate service uptake during the early postpartum period and its predictors in SSA countries. The findings indicate that less than half (42.94%) of women in the region received adequate PNC services. Overall, the adequacy of service uptake use is still too low to accomplish the expected reduction in maternal and newborn mortality and morbidity (24). This might be due to several factors, including a lack of community awareness about the contents of care and their importance, a shortage of healthcare providers, poor road infrastructure, transport challenges, and inadequate medical supplies, all of which are prevailing within the region (25–27). Therefore, governments and other stakeholders in the health system must enhance their efforts to improve both access to and the quality of PNC services. To do this, they should work on increasing community awareness, lowering healthcare costs (by facilitating access to transportation), strengthening home visits, along with enhancing the quality of care at service delivery points and encouraging pregnant women to give birth at health facilities (28–30).

The study examined the influence of factors at the individual and community levels. Accordingly, receiving adequate PNC service was significantly associated with living in a southern sub-region, low acceptance of wife beating, reading newspapers, place of delivery, the timing of ANC, autonomy in decision-making, and quality of ANC.

The subregion in which the women resided was shown to be associated with receipt of adequate PNC, those women in the Southern subregion being more likely to receive it than those in the Central region. This may be partially explained by differences in socioeconomic and cultural conditions that may impede access to healthcare facilities and cause disparities in the use of maternal health services. Given the context of this study, the southern region is represented by South Africa, which is known for its strong economic standing and may account for the uptake of adequate PNC services. This result is consistent with previous studies relating geographic disparities to PNC use in various low-and middle-income countries (15, 31–33).

Timing and quality of ANC were identified as significant predictors of adequate PNC and this was supported by studies conducted in India, Rwanda, Sierra Lion, Zambia, Malawi, and Nigeria (15, 31, 34–37). This could be explained by the fact that if a woman begins prenatal visits early (1st Trimester), she is more likely to receive the desired frequency of visits and counseling sessions. This may result in having enough information about PNC, its value, contents, and when and where to receive it, all of which raise the likelihood that she will receive adequate service (15, 38). Additionally, those women would have easy access to information about potential complications during the postpartum period and their related danger signs, all of which would boost their health-seeking behavior, and raise the possibility of receiving adequate services (39).

Birth at a health facility was also found to be positively associated with receiving adequate PNC service. This was in tandem with studies conducted in Low and Middle-income Countries (40), Indonesia (41), Kenya (42), Uganda (43), and Ethiopia (44). This could be due to healthcare providers informing mothers about the subsequent PPV at home, supported by birth notification following delivery to community health workers to give postpartum care at home visits, as documented by several African countries (44–46). Additionally, they have more chances to get information about the advantages and availability of PNC services at the community level during their stay at maternity wards, which also increases the uptake of adequate PNC (47).

The odds of receiving adequate PNC is found to be higher among women who gave their last birth by Caesarean section(CS) and this was supported by findings of studies conducted in Ethiopia (47). This may be because almost all of the women who underwent CS stayed at a healthcare center for at least 48–72 h, and as reported in many studies, this increased the likelihood that they would receive adequate PNC (44, 48, 49). Furthermore, the women themselves become more wary of postoperative complications, which would also raise their health-seeking behavior. However, it should be stressed that healthcare providers should take into account any potential hazards of CS in the woman’s future life, and they should do so in the context of strong clinical indications (50). In other words, non-medical justifications for CS delivery should be discouraged to the acceptable level advised by WHO (5–15%) (51) to tackle the possible health hazards following the procedure.

Women who exhibited high autonomy in decision-making power had higher odds of receiving adequate PNC services and this was in tandem with studies conducted in LMICs (52), Bangladesh (23), Nepal (53), and Ethiopia (13). This may be because as women attain greater autonomy in their mobility, household purchases, and healthcare decisions, they are less likely to face restraints from their husbands or other family members and are therefore more likely to use MNCH services (54). Women in the SSA region, where a patriarchal society is prevalent, are known to have a gross power imbalance when it comes to maternal and child health service usage because they lack the final say in important decisions that have an impact on their health and well-being (52, 55). Hence, regional and national government agencies have to collaborate in their effort to enhance women’s autonomy through initiatives like education, job opportunities, and finance that boost their ability for independence and decision-making.

When compared to their counterparts, women who were exposed to newspapers and magazines had a greater chance of receiving adequate PNC. Women who have access to this media outlet may also be more well-educated, come from a good socioeconomic background, reside close to health facilities, and have higher levels of health literacy, all of which may increase the likelihood that they will receive adequate postnatal services (15, 34, 56). Thus, media organizations and maternal health service programs should collaborate to ensure that women can access media platforms with adequate and comprehensive maternal health information.

Finally, having a high acceptance toward wife beating was also found to be negatively associated with the receipt of adequate PNC service. This was in line with other studies conducted in Bangladesh, Palestine, Albania, and Ethiopia (20, 57–59). This may be the case because a woman who perceives such violence to be “highly justifiable” will likely be aware of her lessened entitlement, confidence, and status as well as how this may negatively reflect on her sense of empowerment to access maternal health services (60, 61). Violence against women has drastically increased over the past few decades from being seen as a private or family issue to being seen as a social and public health concern with serious effects on health and the uptake of reproductive health services (62). The availability of services is frequently given top priority in most programs and interventions in SSA countries. However, studies indicate that merely having an accessible service scheme does not ensure that women will use it unless the government works to empower women and boost their self-esteem so that they can resist the societal dominance of gender stereotypes (22, 62).

The current study has strengths as well as drawbacks. Firstly, this is the first study in the SSA region to examine the adequacy of PNC services received during immediate PPP by focusing on the contents of care. Secondly, using multilevel mixed-effect models, the effects of individual-and community-level factors on the uptake of adequate PNC have been examined both separately and collectively for better parameter estimates. This is essential for creating effective interventions. Finally, the most recent nationally representative datasets from 18 SSA nations have been used, and this makes it easy to generalize our findings to all women in the region. Despite the aforementioned strengths, there are some limitations in the study. Due to the cross-sectional nature of the study, it can be difficult to deal with social desirability and recall bias and also difficult to establish the cause-and-effect relationship between outcomes and exposures.

## Conclusion

The results showed low coverage of adequate PNC service in SSA countries, indicating that the WHO recommendation for positive postpartum experiences was not implemented satisfactorily. Living in the southern sub-region, institutional delivery, early initiation of ANC, delivery by CS, autonomy in decision-making, attitude towards wife-beating, and reading newspapers were identified as predictors of receipt of adequate PNC service. The Federal Ministry of Health and healthcare managers in each country should enhance their efforts to devise interventions that promote women’s empowerment to enhance their autonomy in decision-making and reduce attitudes towards wife beating. Healthcare providers ought to strive to provide skilled delivery services and early initiation of ANC, along with comprehensive education about the value of receiving adequate PNC service.

## Data availability statement

Publicly available datasets were analyzed in this study. This data can be found at: https://www.dhsprogram.com.

## Ethics statement

The requirement of ethical approval was waived for the studies involving humans because ICF International granted permission. The studies were conducted in accordance with the local legislation and institutional requirements. The participants provided their written informed consent to participate in this study.

## Author contributions

AH: Conceptualization, Formal analysis, Methodology, Project administration, Resources, Software, Validation, Writing – original draft, Writing – review & editing. AT: Formal analysis, Methodology, Writing – review & editing. LT: Methodology, Writing – review & editing.
